# Description of a Case of Hydrocephalus

**Published:** 1797

**Authors:** M. Tenghil

**Affiliations:** Professor of Surgery at Quiers.


XXIII. *Deſcription of a Caſe of Hydrocephalus. By M.* Tenghil, *Profeſſor of Surgery at Quiers.* Vide *Memoires de l'Academie Royale des Sciences. Années* 1790-91., 4to. Turin, 1793.

THE ſubject of the ſingular inſtance of hydrocephalus here deſcribed was a male child, born at Peceto, a hamlet, near Quiers, in Piedmont, on the 21ſt of September, 1790, with a tumour or cyſt hanging down from the back part of the head to the ſhoulders, but without any other apparent defect of ſtructure. The father of the child brought brought it to M. Tenghil three days after its birth, to consult him about this tumour, which was as large as the child's head. Its shape was spherical, and it was connected by a narrow basis, of about an inch in width, to the left side of the occiput. The skin was of its natural colour. Some hairs, which grew on the upper part of the tumour, covered about a third part of it. The veins on its surface were much distended, and there were here and there livid spots, which our author supposed to be the effects of pressure. No very distinct fluctuation, he observes, could be felt. On a suspicion that it might be a case of hydrocephalus, he examined, he tells us, the basis of the tumour, to see if there was any part of the occipital bone wanting; but he was unable to ascertain this point: and as the sutures of the head did not appear to be preternaturally dilated, he candidly acknowledges he was unable to satisfy his mind completely with respect to the precise nature of the tumour. He was convinced, however, that nothing could be attempted for the relief of the child, and that it could not live long; and this opinion he gave to the father.

The child died on the 25th of October, and our our author had then an opportunity of aſcertaining the ſtate of the parts by diſſection.

The ſize of the tumour had increaſed a little from the time he firſt ſaw it. Its length was now rather more than three inches; and, at its largeſt part, it meaſured about ſeven inches and a half in circumference. It was now, however, no longer of a ſpherical ſhape, but was become in ſome meaſure flaccid. Its outer ſurface was excoriated, and covered with a fœtid diſcharge. A fluctuation was now more diſtinctly to be felt. On opening the tumour, a fluid was diſcharged, of a yellowiſh colour, inclining to red, in a greater quantity than the bag itſelf ſeemed to be capable of containing. On the right ſide of the bag, cloſe to the occiput, there was a fungus as large as a walnut; and on touching the upper part of the cavity, contiguous to this ſubſtance, there ſeemed to be a deficiency of bone, but the opening in it was too ſmall to admit the author's finger. He therefore introduced a probe, and found that it paſſed into the cavity of the cranium. He now carefully traced this opening in the os occipitis, and found it to be not more than three or four tenths of an inch in width, and ſituated at the ſide, and a little to the left, of the tuberoſity of of that bone. It was through this opening that a greater quantity of fluid was difcharged than the cyft itfelf was capable of containing. This fluid had preffed in part on the tentorium of the cerebellum, and had fo macerated the left pof-rerior lobe of the brain, that it diffolved on the leaft touch. The middle lobe of the brain, on the left fide, was alfo foftened by it.

The dura mater came out through the opening; but our author, from the macerated ftate of the parts, was unable to afcertain whether it lined the whole inner furface of the bag, or had been torn by the weight of the fluid. He was able, however, to trace the fungous fubftance, through the opening, to its termination a little above it on the furface of the brain, and it feemed to him to have been produced by the cellular texture of the pia mater. The other parts contained within the head were in their natural ftate.

M. Tenghil has contented himfelf with giving, without any comment, this concife defcription of the appearances in queftion, illuftrated by two engraved figures, which our readers will find copied, on a reduced fcale, in Plate II. One of thefe figures (fig. iv.) fhows the external appearance of the bag; and the other other (fig. v.) its internal surface. In this last figure, *a* refers to the opening in the os occipitis, and *b* to the fungous substance above described*.

* We find a case, in some respects similar to this, described and delineated by Job a Meek'ren, in his *Obs. Medico-Chirurg.* p. 53. 8vo. Amstelodami, 1682. See also Ruysch *Obs. Anatomico-Chirurgic. Centur.* (*Obs.* 52. fig. 45) 4to, Amstelod, 1737. Editor.

